# Structure of bovine cytochrome *c* oxidase in the ligand-free reduced state at neutral pH

**DOI:** 10.1107/S2053230X17018532

**Published:** 2018-01-26

**Authors:** Fangjia Luo, Kyoko Shinzawa-Itoh, Kaede Hagimoto, Atsuhiro Shimada, Satoru Shimada, Eiki Yamashita, Shinya Yoshikawa, Tomitake Tsukihara

**Affiliations:** a Picobiology Institute, Graduate School of Life Science, University of Hyogo, 3-2-1 Koto, Kamigori-cho, Ako-kun, Hyogo 678-1297, Japan; b Graduate School of Applied Biological Sciences and Faculty of Applied Biological Sciences, Gifu University, 1-1 Yanagido, Gifu 501-1193, Japan; c KNC Laboratories Co. Ltd, 1-1-1 Murotani, Nishi-ku, Kobe 651-2241, Japan; d Institute for Protein Research, Osaka University, 3-2 Yamadaoka, Suita, Osaka 565-0871, Japan

**Keywords:** cytochrome *c* oxidase, membrane-protein complexes, X-ray structure, neutral pH

## Abstract

Cytochrome *c* oxidase (CcO), the terminal oxidase in cellular respiration, couples proton pumping to O_2_ reduction. Mammalian CcO resides in the inner mitochondrial membrane. Previously, a model of H-pathway proton pumping was proposed based on various CcO crystal structures. However, all previously determined structures were solved using crystals obtained at pH 5.7, which differs from the environmental pH of CcO in the inner membrane. The structures of fully oxidized and ligand-free reduced CcO at pH 7.3 have now been determined. Structural comparison between the oxidized and reduced states revealed that the structural alterations that occurred upon redox change at pH 5.7 in Asp51, the magnesium-containing cluster, haem groups and helix X, which provide important structural evidence for the H-pathway proton-pumping proposal, also occur at pH 7.3. These structural alterations were restricted to a local region of CcO; no domain movement was detected, nor were significant structural alterations detected in peripheral regions at either pH value. These observations indicate that the small and precise structural alterations that occur over the course of the reaction cycle are not affected by pH change, and that isolated CcO precisely performs proton pumping *via* the H-pathway over a wide pH range. Because the pH is not uniform across the molecular surface of CcO, the fact that the overall structure of CcO is not affected by pH changes ensures the high enzymatic efficiency of this protein in the mitochondria.

## Abstract 2

Although the enzymatic activity of cytochrome *c* oxidase (CcO) depends sensitively on pH over a wide range, X-ray structural analyses of bovine CcO have been conducted using crystals prepared at pH 5.7 owing to the difficulty in crystallizing this protein. Here, the structure of ligand-free reduced CcO was successfully determined at 1.99 Å resolution.

## 1. Introduction   

Cytochrome *c* oxidase (CcO), the terminal oxidase in cellular respiration, couples O_2_ reduction to proton pumping. Mammalian CcO is located in the inner mitochondrial membrane and accepts electrons from cytochrome *c* in the intermembrane space (P-side). The protons used for H_2_O synthesis are supplied to the enzyme *via* the D-pathway and K-pathway from the matrix side (N-side), whereas pumping protons are transferred through the enzyme from the N-side to the intermembrane space (P-side) in order to generate a proton-concentration gradient across the mitochondrial inner membrane (Yoshikawa & Shimada, 2015 ▸).

Bovine CcO, a large transmembrane protein, exists as a 420 kDa dimer in its crystalline state. Previously, we reported the structures of four redox-active metal sites [haem *a* (Fe*_a_*), haem *a*_3_ (Fe*_a_*_3_), Cu_B_ and Cu_A_; Tsukihara *et al.*, 1995 ▸] and the whole-protein structure of the oxidized state (Tsukihara *et al.*, 1996 ▸), and noted the structural differences between the oxidized and reduced CcO crystals (Yoshikawa *et al.*, 1998 ▸). The proton-pumping mechanism, later named the H-pathway mechanism, was proposed based on a structural alteration in Asp51 of subunit I upon redox change (Yoshikawa *et al.*, 1998 ▸). The mechanism was refined based on high-resolution X-ray structural analyses of the oxidized and reduced states, as well as reaction-intermediate analogues (Tsukihara *et al.*, 2003 ▸; Aoyama *et al.*, 2009 ▸; Muramoto *et al.*, 2010 ▸; Yano *et al.*, 2016 ▸; Shimada *et al.*, 2017 ▸).

The H-pathway, through which protons are actively transferred, consists of a water channel and a hydrogen-bond network in tandem connecting the N-side to the P-side. The magnesium-containing water cluster, which collects the four protons to be pumped, is linked to the hydrogen-bond network. A structural alteration in helix X between haem *a* and haem *a*_3_ affects the sizes of the cavities in the water channel, resulting in gating of the water channel. The structures of these regions are illustrated in Fig. 3 of a previous paper (Yano *et al.*, 2016 ▸).

The P-side of the molecular surface of CcO is surrounded by the medium (pH 7.3), whereas the N-side resides in the matrix (pH 7.9) (Llopis *et al.*, 1998 ▸). However, all previously reported structures of CcO were determined using crystals formed at pH 5.7. We recently determined the structure of oxidized CcO crystallized at neutral pH to assess the relevance of CcO structures solved at pH 5.7 and to better understand the mechanism of the reaction that occurs under physiological conditions (Luo *et al.*, 2017 ▸). In this study, we determined the structure of a reduced CcO crystal at neutral pH in order to compare the structural alterations that occur upon redox change at neutral pH and pH 5.7.

## 2. Materials and methods   

### 2.1. Preparation of CcO crystals in the fully reduced state   

CcO crystals in the fully oxidized state were prepared as described previously (Luo *et al.*, 2017 ▸). Reduced crystals were prepared by reducing oxidized crystals obtained under slightly different buffer conditions to those used previously (Luo *et al.*, 2017 ▸). After CcO had been diluted tenfold in 25 m*M* Tris–HCl buffer pH 7.3 containing 20 m*M* sodium acetate and 0.35%(*w*/*v*) fluorinated octylmaltoside (FOM), the protein was concentrated to 100 mg ml^−1^ and mixed with polyethylene glycol (PEG) 1500 at a final concentration of ∼3% for batch-wise crystallization. The resultant crystals were equilibrated with the same cryoprotectant solution as used previously for oxidized crystals (Luo *et al.*, 2017 ▸). The crystals were reduced by soaking them for 90 min in 25 m*M* Tris–HCl buffer pH 7.3, 20 m*M* sodium acetate, 0.20%(*w*/*v*) decylmaltoside, 0.35%(*w*/*v*) FOM, 10%(*w*/*v*) PEG 1500, 40%(*w*/*v*) ethylene glycol, 5 m*M* glucose, 1 µ*M* glucose oxidase, 0.5 µ*M* catalase, 7 m*M* sodium dithionate. The reduction of each crystal was confirmed by measuring the visible-light absorption spectrum before flash-cooling in a liquid-nitrogen stream at 100 K.

### 2.2. X-ray diffraction experiments   

All X-ray experiments were carried out on beamline BL44XU at SPring-8 as described previously (Luo *et al.*, 2017 ▸). The absorption spectra of the reduced CcO crystals were measured before and after X-ray diffraction experiments. A total of nine crystals were used to acquire full data sets at a resolution of 1.99 Å. To collect low-resolution data we set up the experimental conditions as follows: X-ray beam cross-section of 20 µm (vertical) × 20 µm (horizontal) at the crystal and a total dose at the sample position of 6.4 × 10^10^ photons s^−1^. The X-ray beam cross-section for collecting high-resolution data was set to 50 µm (vertical) × 30 µm (horizontal) at the crystal; the total dose at the sample was 2.4 × 10^11^ photons s^−1^. The radiation dose for each diffraction experiment was estimated using *RADDOSE*-3*D* (Murray *et al.*, 2004 ▸). Data processing and scaling were carried out using *HKL*-2000 and *SCALEPACK* (Otwinowski & Minor, 1997 ▸). A total of 1019 images were successfully processed and scaled. Structure-factor amplitudes (|*F*|) were calculated using the *CCP*4 program *TRUNCATE* (French & Wilson, 1978 ▸; Weiss, 2001 ▸). Other statistics for the intensity data are provided in Table 1 ▸. Based on comparison of their unit-cell parameters, all crystals at both pH 7.3 and pH 5.7 were expected to be isomorphous.

### 2.3. Structural determination and refinement   

The same procedures as applied for previous structural analyses of CcO crystals obtained at pH 5.7 (Yano *et al.*, 2016 ▸) and oxidized crystals obtained at pH 7.3 (Luo *et al.*, 2017 ▸) were used for structural determination and refinement. Initial phase angles for structure factors up to a resolution of 4.0 Å were calculated by the molecular-replacement (MR) method (Rossmann & Blow, 1962 ▸) using a fully oxidized structure determined at 1.8 Å resolution (PDB entry 1v54; Tsukihara *et al.*, 2003 ▸). The phases were extended to a resolution of 1.99 Å by density modification (Wang, 1985 ▸) coupled with non­crystallographic symmetry (NCS) averaging (Bricogne, 1974 ▸, 1976 ▸) using the *CCP*4 program *DM* (Cowtan, 1994 ▸). The resultant phase angles (α_MR/DM_) were used to calculate the electron-density map (MR/DM map) with Fourier coefficients |*F*_o_|exp(*i*α_MR/DM_), where |*F*_o_| is the amplitude of the observed structure factors. To compare the crystal structure with that of the oxidized state (PDB entry 5xdq; Luo *et al.*, 2017 ▸), we calculated a difference electron-density map using Fourier coefficients (|*F*_oxi_| − |*F*_red_|)exp(*i*α_MR/DM,red_), where |*F*_oxi_| and |*F*_red_| are the observed structure-factor amplitudes of the oxidized and reduced crystals, respectively, both at pH 7.3, and the phase angles α_MR/DM,red_ are the phases of the reduced crystal. Similar difference electron-density maps with coefficients (|*F*_oxi,pH5.7_| − |*F*_oxi_|)exp(*i*α_oxi,pH5.7_) and (|*F*_red,pH5.7_| − |*F*_red_|)exp(*i*α_red,pH5.7_) were calculated to compare the structures obtained at pH 7.3 and pH 5.7 for both the oxidized and reduced crystals, where |*F*_oxi,pH5.7_| and α_oxi,pH5.7_ are the observed structure-factor amplitudes and phase angles, respectively, of the oxidized crystal at pH 5.7 previously determined at 1.5 Å resolution (Yano *et al.*, 2016 ▸) and |*F*_red,pH5.7_| and α_red,pH5.7_ are those of the reduced crystal at pH 5.7 determined at 1.6 Å resolution. A difference electron-density map was calculated with coefficients (|*F*_red,pH7.3_| − |*F*_c,pH7.3_ *|*)exp(*i*α_red,pH7.3_) to inspect contamination by oxidized CcO in the reduced crystal.

## 3. Results and discussion   

### 3.1. Acquisition of intensity data   

Crystal data and intensity data statistics are given in Table 1 ▸. The unit-cell parameters were very similar to those of the oxidized crystal, with deviations of ∼0.1%, which are comparable to those for crystals obtained at pH 5.7 (Yano *et al.*, 2016 ▸). This observation indicates that the CcO molecules in the oxidized and the reduced crystals were very similar in terms of their structures, which affect the molecular packing within the crystals. The unit-cell parameters of the CcO crystals obtained in each oxidation state exhibited a ∼0.7% deviation between pH 7.3 and pH 5.7, which is greater than the deviation between the two oxidation states at the same pH. This observation indicates differences in the molecular packing of CcO crystals at pH 7.3 and pH 5.7, which are affected by the respective natures of the molecular surface. The statistics *R*_merge_, *R*_p.i.m._ and *I*/σ(*I*), completeness and average multiplicity indicate that the intensity data are of high quality at a resolution of 1.99 Å. Average radiation doses ranged from 0.24 to 0.25 MGy for any rotation angle.

### 3.2. Structure determination and refinements   

Structure-refinement statistics are provided in Table 2 ▸. The refinements converged well to *R*_work_ = 0.182, *R*_free_ = 0.202, an r.m.s.d. on bond lengths of 0.0087 Å and an r.m.s.d. on bond angles of 1.25°. Of 3614 amino-acid residues, 72 were not built into the electron-density map owing to poor electron density. In the reduced crystal, 90 residues were in multiple conformations. We assigned 34 lipid molecules, 14 detergent molecules, 1938 water molecules and 33 ethylene glycol molecules in the crystal structure. The average *B* factors of protein atoms were estimated for two crystallographically independent monomers *A* and *B*, which consist of subunits *A*–*M* and *N*–*Z*, respectively, according to the PDB definition. The average *B* factors for monomers *A* and *B* were 41.2 and 52.9 Å^2^, respectively. Because the structures of both monomers in the same crystal were refined under NCS restraints, and monomer *A* (with the lower *B* factor) had higher quality atomic coordinates than monomer *B*, we chose monomer *A* to describe the structural features of CcO.

A peroxide anion detected at the dioxygen-reduction centre (Supplementary Fig. S1) indicates that the reduced crystal contains oxidized CcO at a ratio of 0.20.

### 3.3. Structural alterations coupled with redox changes in metal centres are restricted to the region around the magnesium-containing water cluster, as is the case at pH 5.7   

**Figure 1:**
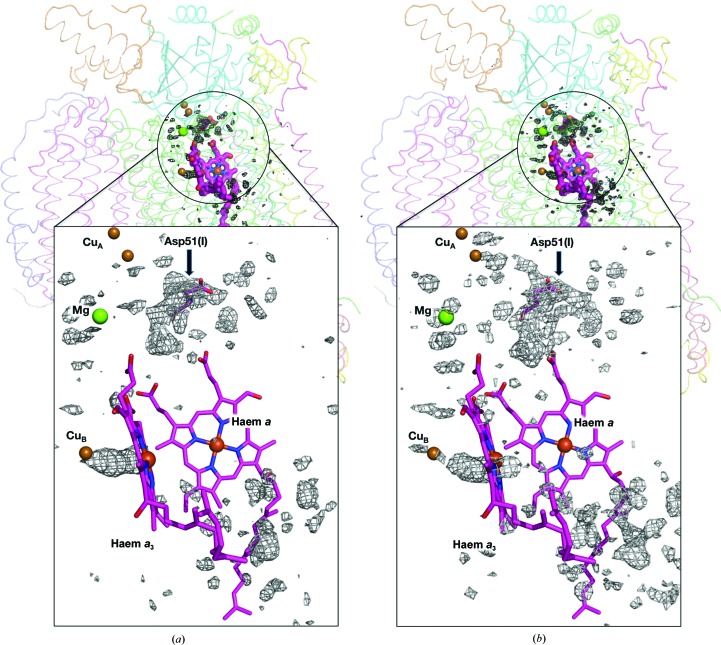
Electron-density differences induced by the reduction of bovine heart CcO are revealed by superposition of (|*F*_oxi_| − |*F*_red_|) difference electron-density maps onto a CcO monomer. The metal cofactors, which contain iron, copper, magnesium and zinc, are displayed as spheres. Asp51 is shown in sticks and indicated by a black arrow. Pink sticks depict haems. (*a*) Difference map of the crystal obtained at pH 7.3 calculated at 1.99 Å resolution. The positive electron-density differences at the 6.0σ level (1.0σ = 0.2308 e^−^ Å^−3^) are shown in black. (*b*) Difference map of the crystal structure obtained at pH 5.7 calculated at 1.6 Å resolution. Positive electron-density differences at the 7.0σ level (1.0σ = 0.2700 e^−^ Å^−3^) are shown in black. The difference map for pH 7.3 shows smaller differences than that for pH 5.7. This is because the reduced crystal at pH 7.3 contains oxidized CcO in part as stated in the text.

The (|*F*_oxi_| − |*F*_red_|) map calculated at 1.99 Å resolution shows the difference in density between the oxidized and the reduced states superposed on the structure of the whole monomer in the oxidized state (Fig. 1 ▸ *a*). The significant structural alterations that occurred following the redox change were restricted to the region around the magnesium-containing water cluster, whereas no other significant difference density was observed in peripheral regions. The similarity between the difference maps at pH 7.3 (Fig. 1 ▸ *a*) and pH 5.7 (Fig. 1 ▸ *b*) strongly suggests that the previously detected structural alterations following the redox change at pH 5.7 are reproduced at pH 7.3.

**Figure 2:**
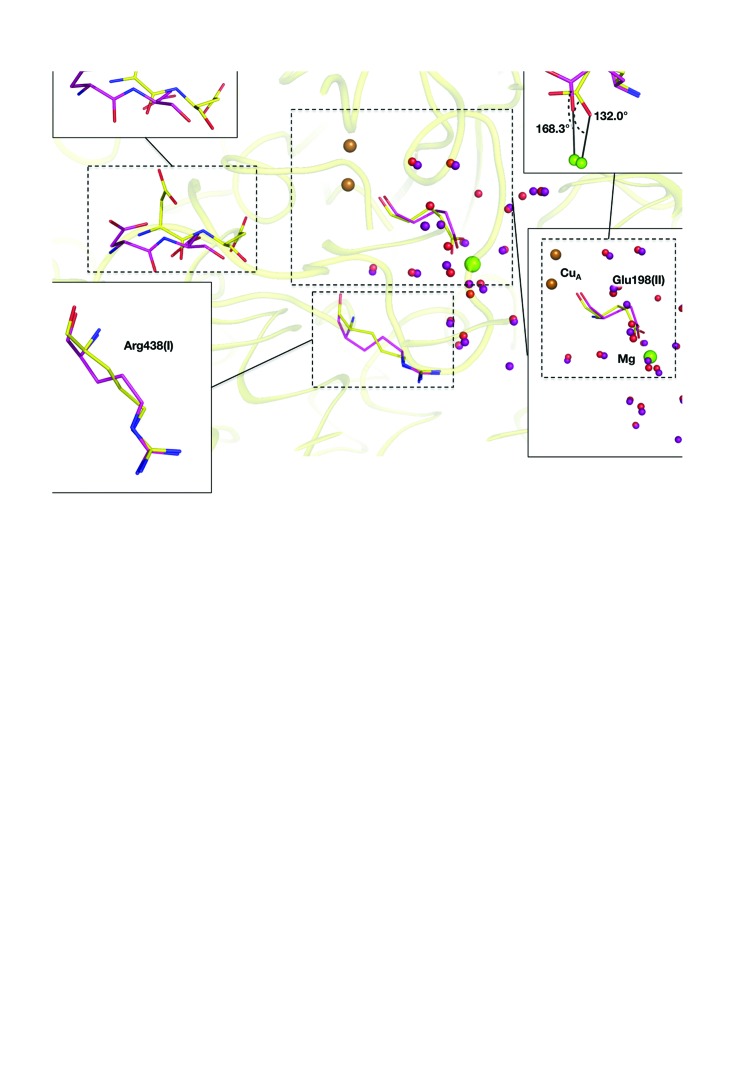
(*a*) Structural differences between the two oxidation states shown as ball-and-stick models. N and O atoms are shown in blue and red, respectively. Green and brown spheres indicate magnesium and copper ions, respectively. Protein C atoms are in yellow and pink for the oxidized and the reduced forms, respectively. Red and purple spheres indicate waters in the oxidized and the reduced states, respectively. Asp51 moves towards the molecular surface upon reduction of the metal centres. Glu198 bridges Cu_A_ and the magnesium ion, and changes its coordination angle upon the redox change. (*b*) Structures of haem *a*, haem *a*_3_ and helix X of the oxidized and reduced states superposed by least-squares fitting of the main-chain atoms of subunit I (subunit *A* in the PDB structure) are shown as stick models, as in (*a*). The structures are at pH 7.3. The capital letters A, B, C and D indicate the pyrrole-ring names according to the PDB definition. Top right: close-up view of hydrogen bonds involving the propionate O atom of the reduced state at pH 7.3. Bottom right: close-up view of close contacts of the terminal part of the hydroxylfarnesylethyl group and amino-acid residues of the reduced state at pH 7.3. A black line indicates close contact within 4.0 Å between an atom of the hydroxylfarnesylethyl group and an amino acid.

Fig. 2 ▸(*a*) shows the structures around the magnesium-containing water cluster in the oxidized and reduced states. Upon reduction of the metal centres, Asp51 of subunit I [Asp51(I)] moves towards the outside of the molecule. The side chain of Arg438(I), a proton-accepting residue in the magnesium-containing water cluster, changes its conformation *via* a pedal motion. Glu198(II) bridges the Mg and Cu_A_ coordinates to Mg *via* its carboxyl group, with C—O—Mg angles of 132.0 and 168.0° in the oxidized and reduced forms, respectively. The conformational transitions upon redox change at pH 7.3 were consistent with those observed at pH 5.7 (Yano *et al.*, 2016 ▸).

A total of 22 sites in the magnesium-containing water cluster were assigned as waters in the MR/DM and (|*F*_o_| − |*F*_c_|) maps. Two sites were within 1.24 Å of each other, as previously observed in the oxidized crystal (Luo *et al.*, 2017 ▸). The two sites were assigned as multiple sites that did not coexist in a molecule, as in the oxidized crystal. All water sites in the magnesium-containing water cluster were in almost the same positions as in the reduced state at pH 5.7. Subsequent to our previous studies (Yano *et al.*, 2016 ▸; Luo *et al.*, 2017 ▸), we determined the occupancies of water molecules in the magnesium-containing water cluster and found that the total number of water molecules was 20.40 (Table 3 ▸); this number is identical within experimental error to that in the oxidized crystal (21.15). These results are consistent with those obtained at pH 5.7, indicating that waters are preserved within the magnesium-containing cluster throughout the reaction cycle.

Pyrrole rings C and D of haem *a*_3_ moved more than pyrrole rings A and B of the same haem upon the redox change, as was the case at pH 5.7 (Yano *et al.*, 2016 ▸). The positions of the propionate attached to pyrrole ring A and the end of the hydroxyfarnesylethyl group were not altered during the redox change. The distances between the two O atoms of the propionate group (O1A in the PDB nomenclature) and the side chain of Asp364(I) (OD1 in the PDB nomenclature) are 2.58, 2.46, 2.52 and 2.45 Å for the oxidized form at pH 7.3, the reduced form at pH 7.3, the oxidized form at pH 5.7 and the reduced form at pH 5.7, respectively. These short interatomic distances indicate that a strong hydrogen bond exists between the propionate of haem *a*_3_ and the carboxyl group of Asp364(I). Furthermore, the propionate group formed a hydrogen bond to His368(I) in all crystals at both pH 7.3 and 5.7. The propionate group is tightly fixed by these hydrogen bonds during the redox procedure under both pH conditions, as shown in Fig. 2 ▸(*b*). The terminal C atoms of the hydroxy­farnesylethyl group of haem *a*_3_, C15–C25, came into close contact with residues belonging to helices VIII and IX of subunit I and helices I and II of subunit II. The residues interacting with the terminal C atoms within 4.0 Å were Ala313 (I), Gly317(I) and Val320(I) of helix VIII; Thr349(I), Gly352(I), Ile353(I) and Ile356(I) of helix IX; Ile34(II) of helix I; and Pro69(II) of helix II (Fig. 2 ▸ *b*). Owing to these interactions, one side of the haem (pyrrole rings A and B) shifts less than the other side (pyrrole rings C and D) upon the redox change at both pH values. The positional shifts of haem *a*_3_ caused by oxidation of the metal centres at pH 7.3 at Fe*_a_*_3_ and a vinyl C atom of CAC (PDB nomenclature) of haem *a*_3_ are 0.35 and 0.67 Å, respectively. These shifts are slightly smaller (by ∼0.10 Å) than the corresponding shifts at pH 5.7. However, the shift of the vinyl group at pH 7.3 is large enough to cause the conformational transition of helix X upon oxidation of the metal centres, as described below.

**Figure 3:**
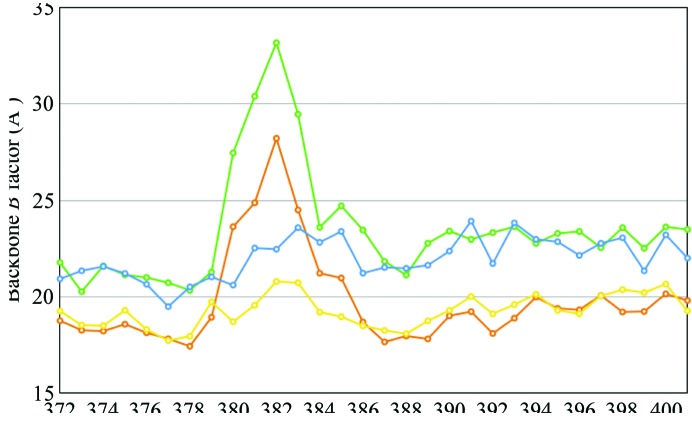
Distribution of average *B* factor along the sequence of helix X. The value for each residue was estimated by averaging those of the main-chain atoms. Blue and green lines correspond to the oxidized and reduced states at pH 7.3, respectively; yellow and brown lines correspond to the oxidized and reduced states at pH 5.7, respectively. In the reduced state under both pH conditions, residues 380–383 have higher *B* factors than the other residues of helix X.

Helix X of the oxidized and the reduced states at pH 7.3 superposed well onto those of the corresponding states at pH 5.7. The structures of helix X in the oxidized and reduced states were termed ‘closed’ and ‘open’ structures, respectively, in our previous paper (Yano *et al.*, 2016 ▸). The translational shift of haem *a*_3_ upon oxidation of the metal centres approached a vinyl C atom of CAC, within 2.76 Å of the C^β^ atom of Leu381(I), in the reduced state. This close contact created a conformational transition of helix X from the open form to the closed form, as was the case at pH 5.7 (Yano *et al.*, 2016 ▸). The *B*-factor distributions of helix X are shown in Fig. 3 ▸. Interestingly, the *B* factors of residues 380–383 in the reduced states at both pH values are significantly higher than those of the other regions of helix X. In contrast, the oxidized structures at both pH values exhibit smooth distributions of *B* factors throughout helix X. These *B*-factor distributions indicate that residues 380–383 are highly flexible in the reduced state. In addition, residues 380–383 are located between haem groups *a* and *a*_3_, and Leu381 closely contacts the vinyl groups of both haems. The closest distances between two haems are 7.35 and 7.61 Å for the oxidized and reduced states at pH 7.3, respectively. Because the space between the vinyl groups of haem *a* and haem *a*_3_ is wider in the reduced state than in the oxidized state, residues 380–383 of helix X are likely to be more flexible in the reduced form than in the oxidized form.

All of the structural differences between the two oxidation states observed at pH 5.7, which provide key evidence for the H-pathway proton-pumping mechanism (Yano *et al.*, 2016 ▸), were reproduced at pH 7.3. Therefore, it is reasonable to consider the H-pathway proton-pumping mechanism as a valid model for the reaction mechanism at neutral pH. The structural alterations upon the redox change are restricted to a local region of the CcO. Consequently, no domain movement occurs, and no significant structural alteration was detected in peripheral regions under either pH condition. These observations indicate that the precise, small-scale structural alterations along the reaction cycle are not affected by pH change, and that CcO performs proton pumping through the H-pathway at a wide range of pH values, which is consistent with the fact that isolated bovine CcO is enzymatically active over a wide pH range (4.6–8.6; Wilms *et al.*, 1980 ▸). This is important given that the pH is not constant across the entire molecular surface of CcO. The stability of the protein structure with respect to pH change ensures that the enzyme preserves high enzymatic activity over a wide range of pH.

## 4. Conclusion   

The structure of reduced bovine CcO crystallized under neutral conditions (pH 7.3) was determined at 1.99 Å resolution. The structural alterations detected at pH 5.7 to Asp51, the magnesium-containing water cluster, the haems and helix X upon redox change, which provide important structural evidence for the H-pathway proton-pumping mechanism, were reproduced at pH 7.3. The structural alterations associated with the redox change were restricted to a local region of the CcO; no domain movement was detected, nor were any significant structural alterations detected in peripheral regions at either pH value. These observations indicate that the small and precise structural alterations that occur during the reaction cycle are not affected by changes in pH, and that CcO can precisely perform proton pumping through the H-pathway over a wide pH range. Because the pH is not constant over the entire molecular surface of CcO, the stability of the structure of the protein over a wide pH range is critical for its high enzymatic efficiency.

## Supplementary Material

PDB reference: bovine heart cytochrome *c* oxidase in the reduced state at pH 7.3, 5xdx

Supplementary Figure S1.. DOI: 10.1107/S2053230X17018532/tb5123sup1.pdf

## Figures and Tables

**Table 1:** X-ray diffraction data for reduced CcO at neutral pH Values in parentheses are for the highest resolution shell.

Beamline	BL44XU, SPring-8
Oscillation angle (°)	0.5
Resolution (Å)	200–1.99 (2.00–1.99)
Wavelength (Å)	0.9
Space group	*P*2_1_2_1_2_1_
Unit-cell parameters (Å)	*a* = 183.31, *b* = 205.90, *c* = 177.53
No. of images	1019
No. of crystals	4
Observed reflections	6110812
Independent reflections	453960 (11255)
Multiplicity	13.5 (8.0)
Completeness (%)	98.8 (98.8)
〈*I*/σ(*I*)〉	31.2 (3.15)
Wilson *B* factor (Å^2^)	30.94
*R*_merge_ † (%)	11 (>100)
*R*_p.i.m._ ‡ (%)	2.5 (41.2)

† *R*_merge_ = 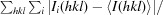 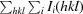, where *I_i_*(*hkl*) is the intensity value of the *i*th measurement of reflection *hkl* and 〈*I*(*hkl*)〉 is the corresponding mean value of *I_i_*(*hkl*) for all *i* measurements. The summation is over reflections with *I*/σ(*I*) greater than −3.0.

‡ *R*_p.i.m._ = 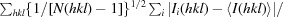 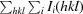, where *N*(*hkl*) is the multiplicity of reflection *hkl*.

**Table 2:** Statistics of structure refinement for reduced CcO at neutral pH

Resolution (Å)	200–1.99
*R*_work_ † (%)	18.22
*R*_free_ ‡ (%)	20.24
R.m.s.d.§, bond lengths (Å)	0.0087
R.m.s.d., bond angles (°)	1.259
Average *B* factors (Å^2^)	
Protein atoms	
Molecule *A*	41.2
Molecule *B*	52.9
Heavy atoms	
Molecule *A*	31.2
Molecule *B*	41.8
Lipid and detergents	79.8
Waters	60.8
All atoms	49.4
No. of amino acids	
All residues	3614
No. determined	3542
No. with multiple conformation	90

† *R*_work_ is the conventional crystallographic *R* factor; *R*_work_ = 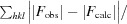 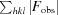, where *F*_obs_ and *F*_calc_ are the observed and calculated structure factors, respectively. Reflections with *F*_obs_ > 0 were included in the refinement.

‡ *R*_free_ is the free *R* factor for the 5% of reflections that were excluded from the refinement.

§ Root-mean-square deviation.

**Table 3:** Occupancies and *B* factors of O atoms in the magnesium-containing water cluster of reduced CcO

Name	No./Chain ID	*B* factor (Å^2^)	Occupancy
1	707/*A*	27.47	1
2	824/*A*	26.03	1
3	840/*A*	30.48	1
4	834/*A*	26.89	1
5	813/*A*	27.82	1
6	863/*A*	29.37	1
7	407/*B*	29.22	1
8	538/*B*	24.51	1
9	418/*B*	27.75	1
10	735/*A*	28.42	1
11	888/*A*	27.21	1
12	821/*A*	26.99	1
13	811/*A*	27.60	1
14	734/*A*	29.25	1
15	737/*A*	29.98	1
16	758/*A*	28.84	1
17	451/*B*	27.46	0.95
18	436_A/*B*	25.83	0.60
19	436_B/*B*	26.37	0.40
20	805/*A*	25.98	0.60
21	415/*B*	28.36	0.95
22	431/*B*	27.36	0.90
Total			20.40
