# Supplementary material for: Structure of bovine cytochrome c oxidase in the ligand-free reduced state at neutral pH
Source: Acta Crystallogr F Struct Biol Commun. 2018 Jan 26;74(Pt 2):92–8. doi: 10.1107/S2053230X17018532 (PMC5947679; doi:10.1107/S2053230X17018532)
Supplement: Supplementary file 1 [file f-74-00092-sup1.pdf]

STRUCTURAL BIOLOGY COMMUNICATIONS

Volume 74 (2018)

Supporting information for article:

Structure of bovine cytochrome *c* oxidase in the ligand-free reduced state at neutral pH

Fangjia Luo, Kyoko Shinzawa-Itoh, Kaede Hagimoto, Atsuhiro Shimada, Satoru Shimada, Eiki Yamashita, Shinya Yoshikawa and Tomitake Tsukihara

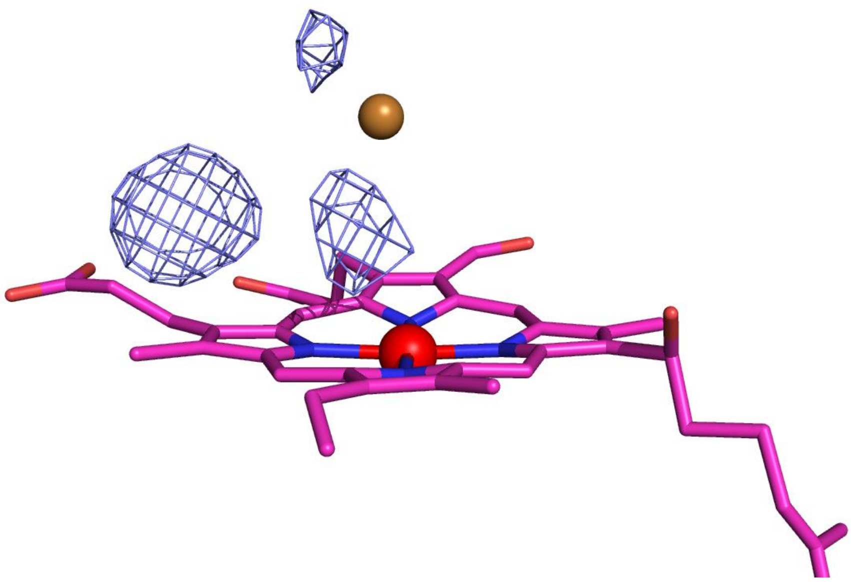

Figure S1 Difference maps calculated with coefficients (|*F*o| - |*F*c|)exp($i\alpha_c$). Heme *a*3 are drawn as stick models, with C, N and O atoms coloured purple, blue and red, respectively. Fe_{a3} and Cu_B are shown as red and brown spheres, respectively. All difference maps were drawn at the 2.0 σ level. The electron density between Fe_{a3} and Cu_B indicates the density of a peroxide anion of the fully oxidized CcO. The large density between two propionates is the density of a water molecule that is not included in the structure refinement. The peak height of the peroxide is 33% of that of the water. The ccupancy of the peroxide converged to 0.20 in the structure refinement. Thus the present reduced crystal contains the fully oxidized CcO at 20%.
